# Hotspots-based patrol route optimization algorithm for smart policing

**DOI:** 10.1016/j.heliyon.2023.e20931

**Published:** 2023-10-16

**Authors:** Dongyeon Kim, Yejin Kan, YooJin Aum, Wanhee Lee, Gangman Yi

**Affiliations:** a Department of Artificial Intelligence, Seoul, 04620, Korea; b Department of Multimedia Engineering, Dongguk University, Seoul, 04620, Korea; c Department of Police Administration, Dongguk University, Seoul, 04620, Korea; d Department of Transdisciplinary Security, Dongguk University, Seoul, 04620, Korea; e Division of AI Software Convergence, Dongguk University, Seoul, 04620, Korea

**Keywords:** Genetic algorithm, Patrol route optimization, Hotspots policing, Police science

## Abstract

Smart policing based on the analysis of big data ensures the development and sustainability of police policy. However, it is difficult to find instances in which the results of data analysis have been applied to actual policy in the field of crime prevention. The South Korean police force recognizes the need for smart policing and is engaged in various research and field support activities. Some examples that are especially relevant for crime investigation include analyzing the connections between cases and predicting the location of the next crime in a series of crimes and the location of suspects. However, it is difficult to find examples of police policy that use big data. Therefore, this study aims to suggest a model that uses big data to respond to emergency calls efficiently. First, we extract hotspots that are predicted to be locations of criminal activity based on an analysis of the association between community environment data and crime data. Second, we create a route having the shortest travel time to the crime location by developing a route optimization algorithm. Lastly, we assess the performance of the patrol routes in reflecting real-time traffic information. If the data application model suggested in this study could be adjusted and applied to the current police patrol system, the model could be used by each police department effectively.

## Highlights

• Suggest a model that uses big data to respond to emergency calls of crime incident.
• Identify crime hotspots based on an analysis of various community & crime data.
• Develop patrol route optimization algorithm for smart policing.
• Derive results analyzing real-time traffic information together.

## 1 Introduction

The concept of predictive policing involves the deployment of police in high-crime areas. This requires the police to be careful when identifying locations with high crime rates, and choosing a specific place to patrol is the most important factor in predictive policing. The manual processes of hotspots policing and patrol route creation have dominated crime prevention initiatives in Korea. Increases in police personnel have brought about meaningful achievements, but to better respond to the increasing diversification and specialization of crime and improve security capacity, science-based strategies must be integrated into police activities. Effective allocation of limited police resources, such as personnel and equipment, is a major concern for governments. It is more urgent than ever to identify and concentrate policing in crime-prone areas for effective crime prevention. Since the 1990s, “hotspots policing” has been in the limelight in many countries, including the United States, as a scientific approach to public security efforts. Numerous empirical studies have shown that police activities in hotspots, which are small geographical areas with high crime rates, can lead to a significant decrease in crime and disorder [1], [2], [3], [4], [5].

In South Korea, academics and police authorities have expressed great interest in hotspots policing, but little is known about how local stations enforce police activities in hotspots, and there is a dearth of empirical studies that test the effectiveness of such strategies. Of course, it should be considered that unlike other countries, such as the United States, detailed regional crime statistics are not disclosed in Korea, so that relevant empirical research is difficult to conduct without the direct assistance of law enforcement.

In this context, a patrol route algorithm that applies advanced digital technologies such as big data analysis can provide a blueprint for the police to respond swiftly and effectively to crime scenes. To date, existing police patrol patterns have largely relied on the personal experiences of on-site police officers and the intuition of the commaning officer. Personal experience and intuition are limited in that they vary widely among individuals, making it difficult to predict policy continuity or expand development potential. Predictive policing, which utilizes data analytics, provides an alternative to overcome these limitations. Thus, this study aims to break away from traditional patrol methods by analyzing crime data and other public data to predict crime risk and occurrence by region and to develop generalized optimal patrol route generation models, thereby enabling proactive policing. The contributions of the proposed approach can be summarized as follows.

• Propose a generalized Smart Policing model with a computational approach that can quickly respond to emergency calls regarding criminal incidents and utilize police resources efficiently.
• Devise a method to estimate the crime risk values of a grid-scale area using correlation analysis between crime statistics and various community data over multiple years.
• Define crime hotspots based on estimated crime risk values and propose patrol route optimization algorithm for efficient hotspots policing.
• Validate the efficiency of proposed routes against common routes by comparing cumulative travel times to the incident location that reflect real-time traffic information.

## 2 Hotspots policing in South Korea

Locations that are the setting of frequent crimes are referred to as hotspots, but the definition of the term differs by scholar. Hotspots can refer to micro-areas or locations where crime is concentrated [6] or locations where crime occurs repeatedly [7]. In South Korea, the term hotspots is used to refer to locations that are prone to or vulnerable to crime.

Crime-prone locations are areas with a high frequency of crimes according to crime statistics, and locations vulnerable to crime are areas where there are worries about crime occurring from the perspective of fear. These terms are therefore similar to the meaning of hotspots but with slight differences in nuance. There is no official definition of hotspots that is agreed upon, but hotspots can be defined as “micro-areas or specific locations where crime occurs intensively or constantly at high frequency” because the characteristics of crime hotspots not only include a high frequency of crime but also include concentrated and consistent crime within a small area. The idea of hotspots originates from theories that suggest the importance of location in understanding crime [8], [9]. The dynamics, circumstances, and attributes inherent in a place are considered to be major factors that explain crime clusters [10]. Locations that are considered hotspots therefore trigger opportunities for crime or facilitate the occurrence of crime [11].

Many researchers have recognized that crime is generally concentrated in certain regions or administrative areas. Through the development of information technology and geographic information systems, it is possible to identify specific crime-prone locations more accurately. Police have learned from experience that crime tends to be concentrated in certain regions or areas, and they have paid more attention to those areas by focusing police activities there to prevent crime. However, there are clear differences between hotspots recognized by experience and those identified through scientific statistical methods using crime data [12].

With the rapid development of science technology and information technology, it is now possible to specifically and accurately identify areas or places where crimes occur frequently. According to some studies, around 50% of the crime in a city occurs in specific micro-areas that constitute approximately 5% of the area of the city [13], [14].

The South Korean National Police Agency has operated the geographic profiling system (GeoPros) since April 2009 by combining various crime data, geographic information systems, and spatial statistical analysis to scientifically prevent crime and support arrest activities. GeoPros performs multiple functions, including the analysis of crime-prone areas, investigation targets, and cases. GeoPros contributes to crime prevention by utilizing the results of crime-prone area analysis to select areas for intensive patrol, to predict areas at risk of having a high crime rate, and to identify sites for CCTV installation. GeoPros is also utilized in investigations to predict the location of major criminals based on spatial statistical analysis.

Nonetheless, predictive models in GeoPros are limited to analyzing criminal cases filed with the police system. This predictive model excludes the relatively minor disputes and safety risks that are not filed as criminal cases among all emergency calls. In other words, there is a clear limit to using GeoPros data as a reference when creating emergency response policies. GeoPros indeed has the function to present analytical data based on continuously updated emergency calls. However, this function is limited to visually displaying previous calls on a map rather than incorporating explanatory variables that may impact emergency calls to predict future risks. If previous calls are analyzed in their unfiltered form, atypical cases such as repeated calls about the same incidents and non-urgent civil complaints are excluded, decreasing the data's reliability as reference material for increasing patrol efficacy.

Hotspots policing has indeed become a renowned police tactic worldwide, but little is known about its operation in South Korea. Studies that examine how hotspots are defined and identified by South Korean police are lacking, as are the type of police tactics used to respond to hotspots, what is recognized as the most effective among such tactics, and whether the police activities conducted conform to hotspots policing (police activities based on science or evidence) as discussed in academia. To date, there has been limited research on producing adequate hotspots policing patrol routes using computational approaches [15]. Furthermore, there is no evaluation of the efficacy of GeoPros developed in South Korea, nor is it known how it is operated in the field. Thus, this research aims to provide a potential solution by presenting a generalized data analytics model that can respond effectively to emergency calls. Unlike the existing manual approaches to selecting patrol routes, which select random spots at which to conduct random patrols or select vulnerable areas to patrol based on the crime rate, this computational approach helps reduce the response time required to arrive at an incident site from any area. It is also highly extensible, as patrol routes can be formed in other regions in the same way simply by changing the target map and data.

The discussion proceeds as follows. First, a computational approach is utilized to derive the results of a correlational analysis between local community variables and data on crime, and map areas that have a high potential crime risk, or hotspots. Second, new patrol routes are suggested based on a route generation algorithm that optimizes the response time required to arrive at an incident site. Third, the effectiveness of these patrol routes is verified by measuring the travel time to the incident point using real-time traffic information of map API. Finally, we describe the contributions, potential applications, and limitations of this project, and we examine avenues for future research.

## 3 Methods

**Figure 1:**
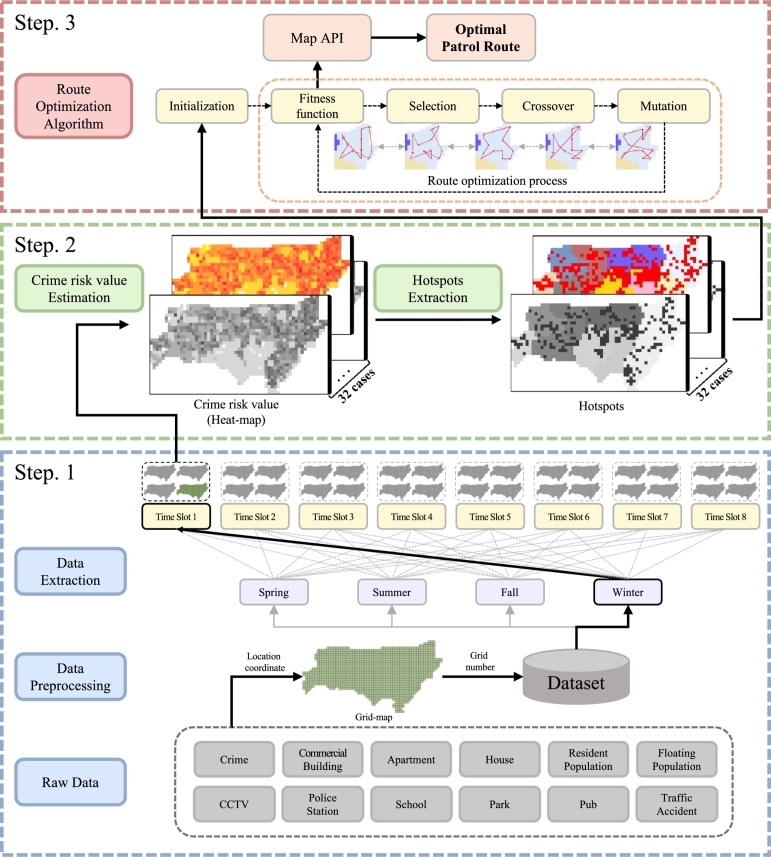
Entire framework of proposed method. In the first step, community data and crime data are collected and preprocessed in a form that can be analyzed. In the second step, the crime risk value for each region is calculated and hotspots are extracted based on the correlation analysis result. In the last step, a patrol route is created through the proposed route optimization algorithm.

In the existing manual approach to local patrol, there is a method for patrolling random spots or spots having a high number of past crimes. However, when a crime occurs somewhere else, the response time may become longer. Therefore, we propose a computational optimization approach to create a patrol route that minimizes the time needed to reach the scene of the crime (Fig. 1).

### 3.1 Data processing

The proposed route optimization algorithm requires diverse and high-quality community data related to crime incidents. In this step, we collect the data and preprocess it so that it can be analyzed according to location and time information. In the case of data that cannot be provided for policy reasons, it is artificially created based on statistical values.

#### 3.1.1 Data collection

The community data that are necessary for data analysis are provided by government departments or private companies. Some of institutions require personal information authentication for data usage, and there are few cities in which data are easy to collect. For these reasons, Seoul, South Korea, which has an authentication process that is easier than those of other countries and has abundant data, is selected as the research area.

Data for Seoul provided by the public data portal of South Korea^1^ is divided into 25 districts and further divided into 425 administrative regions. However, it is difficult to generalize the characteristics of the entire region because the items of data provided for each region are different. Therefore, we select Jung-gu district, located at the center of Seoul, as the research area.

In this research, various items of data that have been proven to be related to crime in research cases about other regions are used [16], [17], [18]. The data items that are collected in the target region are building data (commercial buildings, apartments, houses), population data (resident, floating population) and local environmental data (schools, parks, pubs, traffic accidents, CCTV, police stations). Building data are provided according to the type of buildings and include information on the location coordinates and the number of households respectively. The population data includes the number of residents in each region and the number of floating populations which represent the foot traffic around the region by time. The other data include coordinate information where each data is located.

#### 3.1.2 Generation of artificial crime data

As a matter of policy to prevent personal information leakage and malicious use, crime data in South Korea cannot be provided. Therefore, in order to generate artificial crime data as similar as possible to actual crime characteristics, we collected and analyzed crime statistics by year, month, time of day, and crime type. The Seoul Open Data Plaza^2^ provides statistics on crime by year, month, and crime type. Moreover, the Korean Statistical Information Service^3^ provides statistics on crime over time. Therefore, the actual crime rate can be calculated based on these data.

We obtained statistical data on the incidence of five major crimes (murder, assault, robbery, rape, theft) in Seoul by year from 2008 to 2019. The statistical data from 2014 to 2019 provide the number of crimes in all districts of Seoul. As a result of calculating the crime rate of Jung-gu among all districts of Seoul, the average rate is found to be about 4%. Based on this ratio, we calculate the number of crimes in Jung-gu from 2008 to 2013 (Table 1).

**Table 1:** Frequency of each of five major crimes by year in Jung-gu, Seoul.

Year	Number of Crime Occurrences		Rate of Crime Occurrences	Average Rate	Calculated Results
	Seoul	Jung-gu			
2008	107,771	-	-	-	4,278
2009	115,752	-	-	-	4,595
2010	124,447	-	-	-	4,940
2011	132,939	-	-	-	5,278
2012	137,725	-	-	-	5,468
2013	132,966	-	-	-	5,279
2014	130,674	5,231	0.040	0.039	-
2015	126,401	4,954	0.039		-
2016	117,839	4,584	0.038		-
2017	107,835	4,184	0.038		-
2018	101,948	4,030	0.039		-
2019	103,668	4,327	0.041		-

**Figure 2:**
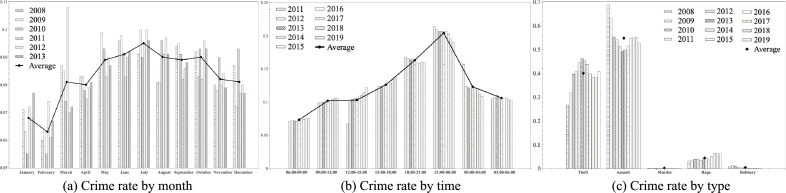
Percentage of crime occurrence by month, time slot, and crime type. In (a), the season of the year having the highest crime rate is from June to August, which is summer in Korea. Conversely, the season with the lowest rate is winter, which corresponds to January and February. In (b), the time slot for the most crimes is from 21:00 to 00:00. The time slot having the lowest incidence of crime is from 06:00 to 09:00. In (c), assault cases are the most frequent type of crime and account at approximately 54.83%. Conversely murder cases account at approximately 0.016%.

To check the monthly rate for each of the five major crimes, an average value is calculated based on the statistical data from 2008 to 2013 (Fig. 2-a). Also, using the statistical data from 2011 to 2019, we can check the crime rate in three-hour units (Fig. 2-b). As a result of calculating the crime rate for each month and time slot, we confirm the fact that crime increases and decreases linearly with the change of season and time. Lastly, based on the statistics from 2008 to 2019, we check the crime rate by crime type (Fig. 2-c).

To reflect the fact that the actual crime rate varies by month and time slot, the date and time of the artificial crime data are based on the calculated average rate (Fig. 2-a, b). The number of generated data samples is set equal to the number of crimes per year (Table 1), and the crime types are designated according to the number of the occurrence of five major crimes (Fig. 2-c). By generating artificial crime data based on the aforementioned statistical analysis, we can accurately analyze the correlation with community data and extract meaningful hotspots.

#### 3.1.3 Data preprocessing

Most of the data represent different geographical ranges and use different data structures for each item. Without preprocessing the raw data, it is difficult to identify the correlation between each data value, so the standardization of data expression methods and grouping rules is required. In this research, data are grouped into grid units based on location coordinates and are expressed as numerical values so that the regional crime risk can be calculated.

The proposed route optimization algorithm generates patrol routes based on hotspots, which represent the grids with a high probability of crime. These hotspots are extracted based on the results of the correlation between crime data and community data in the adjacent area. For the correlational analysis, all items of data included in the same grid are grouped based on the location coordinates of the data.

Most of the collected data represent location information using latitude and longitude coordinates. To group data from adjacent areas, we divide the entire region into equally spaced grids and assign sequential numbers to each grid. To accurately divide the entire region into equal intervals of the smallest size, the 100*m* × 100*m* grid map provided by the National Geographic Information Institute^4^ is used. As a result, 1107 grids are created.

#### 3.1.4 Data extraction

Because crime data include information on the date and time of a crime event, it is time series data in which the occurrence pattern changes according to the passage of time (Fig. 2). Floating population data are also time series data, so the value changes depending on the time slot. Therefore, it is necessary to create a separate model that can be selected by season and time slot to reflect the characteristics of time series data.

**Figure 3:**
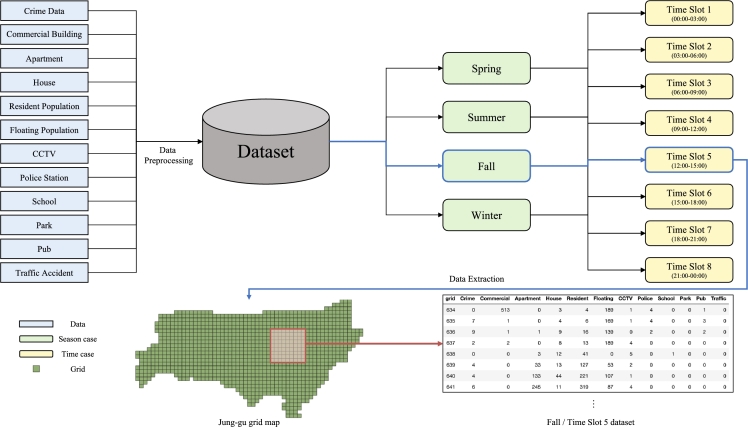
Process for extracting the dataset in each case. After the data are collected, they are preprocessed and stored in the database. It is possible to extract and analyze only the data of one case that corresponds to the time of route creation. This example shows the data extraction process for the case for Fall × Time Slot 5.

In this study, to represent seasonal changes in the crime rate, the four seasons of South Korea are classified as spring (March–May), summer (June–August), fall (September–November), and winter (December–February). In addition, to represent the crime rate and floating population change by the time, the day is divided into eight time slots. As a result, we create a model in which all the data are classified into 32 cases. The crime risk values of the entire grids for each case are calculated; based on the values, an optimal patrol route is created that reflects the characteristics of each season and time slot (Fig. 3).

### 3.2 Determination of crime risk area

Because hotspots are used in the initial route creation and route optimization step, the performance of the route is affected by the definition of hotspots. In the proposed method, hotspots are defined according to the crime risk value estimated by simultaneously reflecting the correlation analysis results and the data value.

#### 3.2.1 Crime risk value estimation

To estimate the crime risk value for each area, we calculate the crime risk value $R_{i}$ of grid *i* using Eq. (1). According to the proposed crime risk estimation method, even areas that have a similar cumulative number of crimes may have different crime risk values. In this equation, the Pearson correlation method is used to consider the correlation between crime data and community data. Using this method, we can analyze the variation of each community data item in response to changes in crime data values and represent the measure of association with crime as a numeric value. In addition, because the range of values expressed for each item of data is different, normalization is performed for each item so that the relative value of data can be considered.

(1) $$R_{i}=\Sigma_{t=1}^{t=T}c_{t}\frac{d_{t,i}}{\Sigma_{k=1}^{k=N}d_{t,k}},$$

where *T* is the total number of data items. $c_{t}$ represents the correlation coefficient between crime data and the *t*-th data item. $d_{t,i}$ means the value of the *t*-th data item of grid *i* and the denominator represents the sum of all identical data items existing in the entire region for normalization. *N* is the total number of grids. Because $c_{1}$, which is the correlation coefficient of crime data (t=1), is 1, it always has the largest weight value. For other items, $\left|c_{t}\right|$ and $\left|\frac{d_{t,i}}{\Sigma_{k=1}^{k=N}d_{t,k}}\right|$ determine the reflection rate for crime risk calculation, and the sign of $c_{t}$ determines the direction of change in value.

However, because the characteristics of each area are different, when the analysis is performed using the data for the entire region, the characteristics of each area cannot be accurately represented (Table 2). The western area of Jung-gu (Grid No.126) has a small resident population and a large variation in the floating population by time slot. On the other hand, in the eastern area (Grid No.1063), the resident population is concentrated. Therefore, if we analyze the data for the entire region, the characteristics of various areas are mixed. To solve this, we consider only the data for adjacent grids. Because the crime risk is calculated for each grid, correlational analysis is performed by grouping the data that are included in the adjacent grids around the target position. We define the grids included within a radius of about 500*m* from the target grid as adjacent areas. When this method is applied, we can check the results that reflect the characteristics of each area well compared with the results of analysis with the data of the entire region.

**Table 2:** Correlation analysis results.

Categories	Grid	Data Item								
		Resident Household	Floating Population	Commercial Buildings	CCTV	Pub	Police Station	School	Traffic Accident	Park
Value of Each Data	126	2	406	0	1	0	4	0	1	0
	436	0	0	0	0	0	0	0	0	1
	1063	239	0	0	0	0	0	0	0	1

Correlation Coefficient	Entire	0.35	0.34	0.03	0.12	0.13	-0.04	-0.22	0.08	-0.43
	126	0.37(+0.02)	0.45(+0.11)	-0.12(-0.15)	-0.01(-0.13)	0.38(+0.25)	-0.12(-0.08)	-0.23(-0.01)	0.15(+0.07)	-0.31(+0.12)
	436	0.94(+0.59)	0.46(+0.12)	0.00(-0.03)	-0.06(-0.18)	0.00(-0.13)	0.00(+0.04)	-0.10(+0.12)	0.32(+0.24)	-0.65(-0.22)
	1063	0.62(+0.27)	0.44(+0.1)	0.00(-0.03)	0.05(-0.07)	0.00(-0.13)	0.00(+0.04)	-0.40(-0.18)	0.00(-0.08)	-0.48(-0.05)

**Figure 4:**
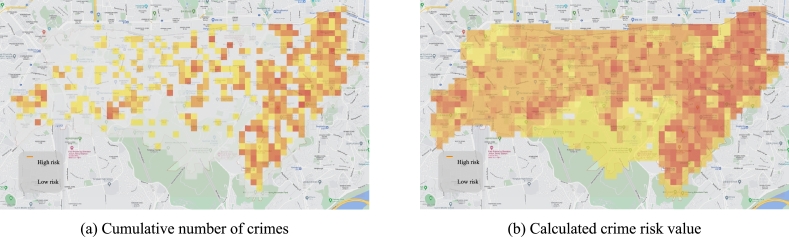
Heat-map of the cumulative number of crimes and the calculated crime risk value. In most cases, a grid with many cumulative crimes in (a) has a high crime risk in (b). Moreover, there are many cases where a grid with a small number of crimes in (a) has a high crime risk value in (b). This is because those grids contain a lot of data that is strongly correlated with crime occurrence.

For all grids, crime risk values that reflect regional characteristics are calculated and can be visually identified in a heat-map. Unlike existing methods that simply define areas with a high cumulative number of crimes as hotspots (Fig. 4-a), the proposed method estimates the relevance of crime to community data representing local characteristics to redefine hotspots (Fig. 4-b).

#### 3.2.2 Hotspots extraction

**Figure 5:**
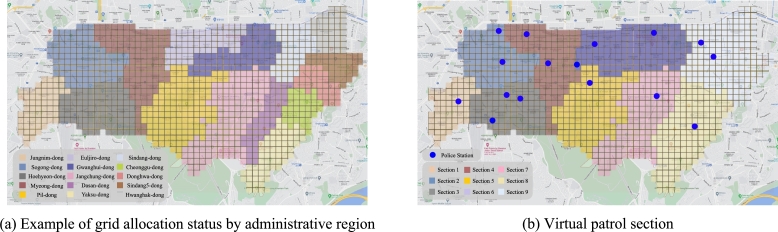
Allocation of patrol sections. (a) represents grid allocation status according to the range of administrative regions. (b) represents virtual patrol section created by combining administrative regions.

There are 15 administrative regions and 16 police agencies in Jung-gu (Fig. 5-a). The size of the patrol section and patrol route is different for each station. Patrol section information for each station is required, but for security reasons, it is not provided. Therefore, in this study, nine virtual patrol sections are formed in consideration of the regional police station status, and geographical and environmental characteristics (Fig. 5-b).

The actual police agency's patrol section and patrol route are smaller than the virtual section and the proposed route. However, the purpose of this research is to create a route that reduces the initial response time relative to existing routes within the same section. Thus, if the performance of the proposed route is verified in the virtual section, time reduction can be expected even in an area of greater subdivision.

**Figure 6:**
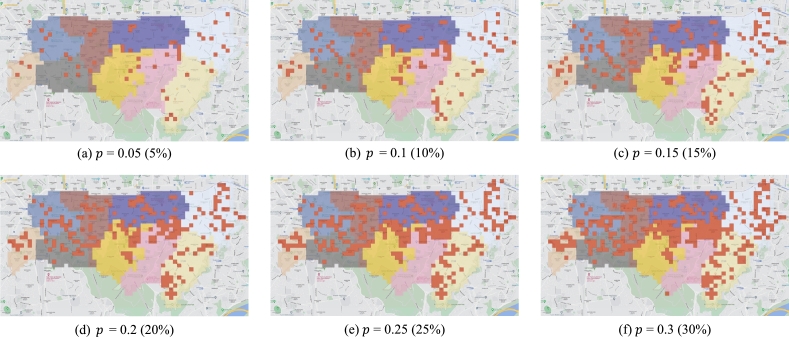
Status of the hotspots according to parameter *p*. Each figure (a)∼(f) is the result of setting the *p* value from 0.05 to 0.3. As parameter *p* decreases, grids having a relatively high risk are defined as hotspots, so a route having a short travel time to the relatively dangerous regions is created, but the travel time increases when a crime occurs in a non-hotspot area. On the other hand, as *p* increases, grids having low risk are also defined as hotspots, so a route is created that considers the entire region, but the travel time to the hotspots may increase.

Because every grid has its own crime risk value, it is possible to extract grids having a relatively high risk in a specific region. According to the extraction parameter, some grids are extracted and defined as hotspots (Fig. 6). If the grids with high risk are extracted sequentially from the entire region, hotspots may be biased in high-risk sections. Therefore, hotspots are extracted for each section by the number of grids calculated through Eq. (2).

(2) $$H_{s(i)}=n_{i}p,$$

where *i* is the section number. $n_{i}$ is the entire number of grids in section *i* and *p* is an extraction parameter that controls the number of hotspots and satisfies 0≤p≤1.

In the proposed route optimization algorithm, we utilize the hotspots information for each section in the waypoint extraction and the fitness function definition step. As such, hotspots have an impact on the performance of the algorithm, so it is necessary to set the optimal parameter *p* to determine the proper number of hotspots. According to the experimental results, when p≤0.2, a relatively simple route is generated due to the insufficient number of waypoints (Fig. 6-a∼d). Furthermore, when p≥0.3, the operation time increases and the performance improvement effect is weak because the hotspots are biased in a specific area (Fig. 6-f). Therefore, to extract multiple hotspots and maximize the performance improvement, we determine *p* to be 0.25 (Fig. 6-e).

### 3.3 Route optimization algorithm

We determine the patrol points based on hotspots for each section and create a route that passes all the points. Because various types of routes are generated according to the order in which the waypoints are passed, we propose an optimization algorithm to find the optimal sequence that reduces the travel time to the hotspots.

This can be thought of as a problem similar to the TSP: traveling salesman problem [19], [20], [21]. In order to solve a general TSP, an optimal route is formed that starts from a specific spot, traverses all the waypoints, and returns to the starting point with the minimal travel time. If the brute force algorithm is applied, the optimal route can be generated because all possible cases can be checked. However, as the number of waypoints increases, the number of calculations increases dramatically. As a result, efficiency is substantially reduced in terms of time and space complexity, and the brute force algorithm cannot be used as an algorithm for generating a route in an actual application.

**Figure 7:**
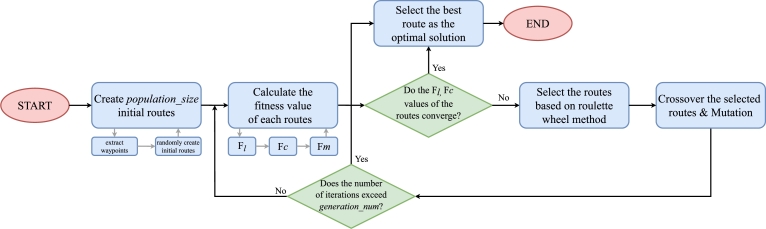
Illustration of the genetic algorithm. The initial route is generated in the initialization step and optimized by repeating selection, crossover, and mutation until the termination condition is satisfied.

To solve this problem, various optimization methodologies such as ant colony optimization [22], [23], [24], genetic algorithm [25], [26], [27], tabu search [28], [29] and particle swarm optimization [30] have been proposed. In our case, we modify some of the existing algorithms because we consider not only minimizing the travel time of the route itself but also reducing the travel time to the hotspots. Specifically, we create an optimal route based on the genetic algorithm, and we include a figure to illustrate the entire optimization process (Fig. 7).

#### 3.3.1 Initialization of the routes

**Figure 8:**
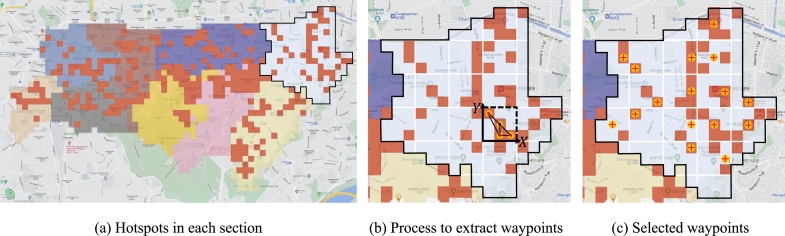
Method for waypoint determination. (a) represents the hotspots of each section. And (b) represents the waypoints extraction process. Each section is divided into subsections of the same size, and the grid located at the average distance to all hotspots in the same subsection is determined to be a waypoint. (c) represents the extraction results.

Like the initialization process for the genetic algorithm [31], [32], the proposed algorithm determines patrol points based on hotspots and creates initial routes (Fig. 8-a). During the process to determine the patrol points, if the centrally located grid from many hotspots in one area is determined as a patrol point, the surrounding areas can be patrolled together. To reflect this, each section is divided into a 3 × 3 grid to form the smallest subsection, and a grid located at an average distance from the hotspots is selected as a patrol point. Also, for subsections where there is no hotspot, patrol points are not extracted because the need to patrol these areas is low (Fig. 8-b, c). The order of passing the extracted waypoints is randomly determined, and as many initial routes are created as the number set by the generation_num, which is one of the basic parameters of the genetic algorithm.

#### 3.3.2 Selection of candidate routes

In the selection step of genetic algorithm, two candidate solutions are selected and combined to generate a new solution that constitutes the next generation's population. These two candidate solutions are selected using a fitness function that calculates a fitness value to represent the performance of each solution [31], [33]. Similarly, in the selection step of proposed algorithm, we define a fitness function that can simultaneously reflect the overall length of the route and the average travel time to the hotspots. After that, the fitness values of all routes existing in the current generation are calculated and two candidate routes are selected by applying the roulette wheel method [32], [34], [35].

The fitness function for solving the TSP is generally defined as the sum of the Euclidean distance between adjacent waypoints, as in Eq. (3), to minimize the length of the path itself [31], [36].

(3) $$F_{l}=\frac{1}{\Sigma_{j=1}^{j=n-1}d_{e}(w_{j},w_{j+1})+d_{e}(w_{n},w_{1})},$$

where *n* represents the total number of waypoints included in the route, and $w_{j}$ is the grid corresponding to the *j*-th waypoint of the route. $d_{e}(w_{j},w_{j+1})$ means the Euclidean distance between the waypoints $w_{j}$, $w_{j+1}$.

However, the proposed algorithm simultaneously considers $F_{c}$ in Eq. (4) to reduce the average travel time to the hotspots. $F_{c}$ means the average distance from each grid that the route passes through to all hotspots in the current section.

(4) $$F_{c}=\frac{rH_{s(i)}}{\Sigma_{k=1}^{k=r}\Sigma_{l=1}^{l=H_{s(i)}}d_{e}(g_{k},h_{l})},$$

where *r* indicates the number of grids that the route passes through, and $g_{k}$ indicates the grid through which the route passes the *k*-th time. $h_{l}$ represents the *l*-th hotspot grid of section *i*.

**Figure 9:**
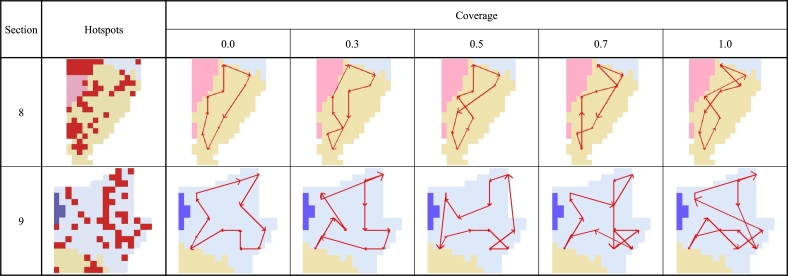
Generated routes according to the *coverage* value. As the *coverage* value decreases, the overall length of the optimized route becomes shorter. Conversely, as the *coverage* value increases, the length of the optimized route becomes longer. The route becomes more complex and passes most of the hotspot grids.

The fitness function of the proposed algorithm, which considers both $F_{l}$ and $F_{c}$ values, is defined in Eq. (5).

(5) $$F_{m}=\frac{(1-coverage)\times F_{l}}{\Sigma_{s=1}^{population\_size}F_{l(s)}}+\frac{coverage\times F_{c}}{\Sigma_{s=1}^{population\_size}F_{c(s)}},$$

where coverage is a weight parameter, and $F_{l(s)}$ and $F_{c(s)}$ represent the $F_{l}$ and $F_{c}$ values of the *s*-th route included in the current generation. We normalize the $F_{l}$, $F_{c}$ value of the route.

coverage determines the reflection ratio of $F_{l}$, $F_{c}$ and affects the shape of the optimized route (Fig. 9). Therefore, it is necessary to determine the coverage value that maximizes performance. We verify the optimal parameter value for all cases in the experimental stage.

When candidate routes are selected based on fitness values, local optimization problems may occur if the two routes having the largest $F_{m}$ are selected from among all routes existing in one generation. To prevent this, the routes are selected probabilistically by applying the roulette wheel method. According to Eq. (6), the $F_{m}$ of each route is converted into a probability that the route is selected. After that, two candidate routes are selected according to the probability value.

(6) $$p_{s}=\frac{F_{m(s)}}{\Sigma_{n=1}^{n=population\_size}F_{m(n)}},$$

where $F_{m(s)}$ represents the $F_{m}$ value of the *s*-th route belonging to the current generation, and $p_{s}$ is the probability value that the corresponding route is selected.

#### 3.3.3 Crossover of candidate routes and mutation

**Figure 10:**
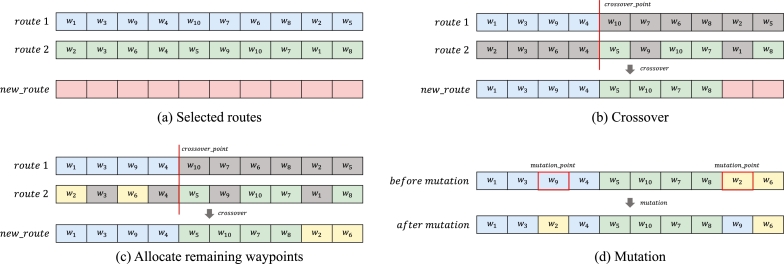
Process of crossover and mutation. Figures (a)∼(c) show each step of the crossover operation, and (d) shows the mutation operation. The routes *route* 1, *route* 2 are candidate routes, and *new*_*route* is a route for the next generation.

In the crossover step, we define a crossover method to combine two candidate routes (Fig. 10-a), and we create a new route. Because a new route is created by combining some of the waypoints included in each route, crossover_point for dividing the routes is designated by an arbitrary point [32], [37], [38], [39], [40]. To preserve a part of route1, the waypoints located at an index smaller than crossover_point are copied to the same location of new_route. In addition, the waypoints located at an index larger than crossover_point in route2 are sequentially allocated to the empty space of new_route. In this process, if a waypoint is already assigned to new_route, we do not reassign it. So, there may be an empty space at the end of new_route (Fig. 10-b). The empty spaces in new_route are filled by sequentially allocating the unallocated waypoints of route2 (Fig. 10-c).

If the route is optimized by repeating the selection and crossover process, $F_{l}$, $F_{c}$ converges to a specific value. However, if the types of routes existing in the same generation are not diverse, a local optimization problem may occur. Therefore, to maintain the diversity of routes existing in a single generation, we apply the exchange technique, which is one of the mutation methods of the genetic algorithm [31], [38], [41]. Two mutation_points are randomly selected from among the waypoints of the new_route and exchanged to determine the final route (Fig. 10-d).

In conclusion, in the initialization step, initial routes as many as population_size are created, and the selection, crossover, and mutation operations are repeated until the number of repetitions determined by generation_num is exceeded, or the values of $F_{l}$ and $F_{c}$ converge. Finally, the route having the largest fitness value is determined to be the optimal route.

## 4 Experiments

In the proposed algorithm, there are four parameters used for route optimization: population_size, generation_num, crossover_rate, and mutation_rate. Because the performance of the route is determined according to the parameter setting, it is important to set the optimal parameter combination [42], [43], [44]. In the experimental step, the parameters of the route optimization algorithm are optimized for 9 sections in 8 time slots that can be tested in the current season. Afterwards, validation of the route according to the coverage value is performed by using real-time traffic information. We verify the superiority of the computational optimization approach by comparing the performance of the route generated using the optimized parameters and the performance of the route that patrols random spots.

### 4.1 Parameter optimization

In the parameter optimization process, in order to evaluate the performance of the route generated by each parameter setting, we compare the $F_{a}$ values calculated using Eq. (7).

(7) $$F_{a}=\frac{\Sigma_{i=1}^{i=iteration}\Sigma_{j=1}^{j=population\_size}(1-coverage)\times F_{l(i,j)}+coverage\times F_{c(i,j)}}{iteration\times population\_size},$$

where iteration indicates the current generation number, and $F_{l(i,j)}$, $F_{c(i,j)}$ refer to the $F_{l}$, $F_{c}$ values of the *j*-th route belonging to the *i*-th generation.

$F_{a}$ represents the average fitness value of the routes in each generation that has been generated up to the current iteration. It performs two evaluations. First, we can compare the speed at which the fitness value of the route converges as the generations increase. Even if two populations generated by different parameter settings finally converge to the same fitness value, the $F_{a}$ value of the population that began to converge in a early generation is larger. In an optimization problem, the faster the fitness value of a solution converges, the fewer calculations are needed, which is advantageous in terms of cost. Second, it is possible to check whether a local optimization has occurred. Even if two populations begin to converge in the same generation, they have a relatively smaller $F_{a}$ value when local optimization occurs. Because local optimization is directly related to the performance of the patrol route, bad parameter combinations should be excluded.

#### 4.1.1 Optimization of generation_num and population_size

**Figure 11:**
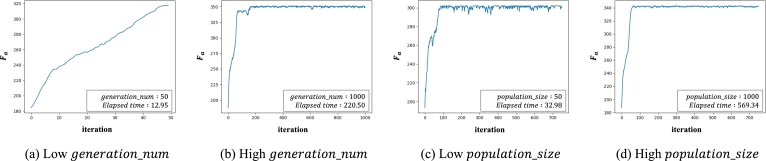
Graph representing the *F*_*a*_ value of each iteration according to the *generation*_*num*, *population*_*size* settings. In both graphs (a) and (b), all conditions are the same except for the *generation*_*num* parameter value. In (a), the optimization algorithm is terminated before the fitness values converge. On the other hand, (b) converges to a relatively large value, but unnecessary calculations are continuously performed. In both graphs (c) and (d), all conditions are the same except for the *population*_*size* parameter value. Although the elapsed time in (c) is short, the graph converges to a relatively low value. However, (d) converges to a high value, but the execution time is significantly increased.

generation_num determines the number of repetitions of the selection, crossover, and mutation operations, and population_size determines the number of routes in one generation. If the value of generation_num is too small, the optimization process ends before the fitness value converges (Fig. 11-a). Conversely, if the value is too large, the process repeats the iteration even though the fitness value converges (Fig. 11-b). If the value of the population_size parameter is too small, there is a high probability of local optimization problems because the number of routes that constitute one population is too small (Fig. 11-c). Conversely, when the value is too large, there is a problem in that the calculation time increases (Fig. 11-d).

In order to find the combination of optimal parameters, a candidate value is designated for each parameter, and three combinations having a large $F_{a}$ value are sequentially selected. We set generationn_num to [50, 100, 200, 500, 750, 1000, 1500] and population_size to [50, 100, 300, 500, 750, 1000], and we compare $F_{a}$ values for all parameter combinations. According to the experimental results, when each parameter exceeds a certain value, the change in $F_{a}$ value is insignificant, so the maximum values are set to 1500 and 1000, respectively. In addition, to remove outliers, the evaluation of each parameter combination is repeated five times, and the averages of the resulting values excluding the maximum and minimum are compared.

#### 4.1.2 Optimization of crossover_rate and mutation_rate

**Figure 12:**
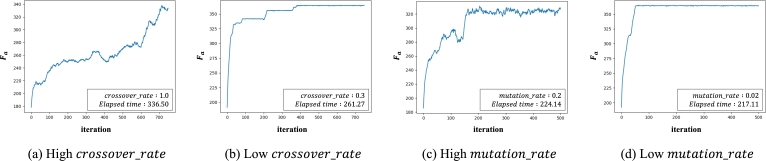
Graph representing the *F*_*a*_ value of each iteration according to the *crossover*_*rate* and *mutation*_*rate* settings. In both graphs (a) and (b), all conditions are the same except for the *crossover*_*rate* value. In (a), the deviation in *F*_*a*_ according to the iteration is very large and the value does not converge. On the other hand, in (b) the graph converges stably. In both graphs (c) and (d), all conditions except *mutation*_*rate* are the same. In (c), mutation occurs relatively frequently, so the deviation in *F*_*a*_ according to the iteration is relatively large. On the other hand, in (d), the graph converges stably to a larger value.

crossover_rate determines the probability of executing the crossover operation on candidate routes. If a crossover operation is performed on all candidates, the convergence of fitness value becomes difficult (Fig. 12-a). On the other hand, if the parameter is set to properly, we can have relatively better results (Fig. 12-b). mutation_rate determines the probability of executing a mutation operation. Setting too large mutation_rate values can make optimization difficult (Fig. 12-c). On the other hand, setting a proper value can prevent the problem of local optimization (Fig. 12-d). To determine each parameter, we set the crossover_rate between 0.1 and 1.0 in units of 0.1, and we set the mutation_rate to [0.0, 0.01, 0.03, 0.05, 0.07, 0.1]. We then compare the $F_{a}$ values for each route.

The optimization process for the four parameters is performed for each of the 72 cases, and the optimal parameter values for each case are determined.

### 4.2 Results

In the experimental step of this research, the ability of the proposed method to reduce the initial response time when an incident occurs is confirmed. First, by comparing the cumulative initial response time for each route according to the setting of the coverage parameter, the time reduction is analyzed, and the optimal parameter setting is determined. Second, the performance superiority of the proposed method is proved through an experiment that compares the cumulative initial response time of the optimal route and the random route.

Before we compare the random route and the proposed route, it is necessary to optimize the coverage parameters. The smaller the coverage value, the smaller the reflection rate of hotspots, and the optimal route becomes similar to the shortest route of the manual approach method. Conversely, as the coverage value increases, the reflection rate of hotspots increases. Therefore, it is possible to verify the superiority of the proposed method by using the parameter optimization results derived from all cases.

In the first experiment, to measure the travel time according to the coverage parameter settings, routes of each case are created using five coverage values, [0.0, 0.3, 0.5, 0.7, 1.0]. Then, the movement time from any point along each generated route to the event point is measured. The artificial crime data for the test were generated 1000 times for each case to perform a sufficient number of repeated experiments. We perform this validation for all cases. To measure the actual travel time to the test point, we need to know our current location and to reflect real-time traffic information. However, because the travel time of the route cannot be measured by actual patrols, the current location is determined in a probabilistic method, and real-time traffic information is reflected through the Navigation API. In order to determine the current location, it is assumed that the longer the travel time between two adjacent waypoints, the higher the probability of being present. Then, using the Navigation API, the travel time between adjacent waypoints is measured and converted to a proportional probability value according to Eq. (8).

(8) $$P_{i,j}=\frac{t_{i,j}}{\Sigma_{i=1}^{i=n-1}t_{i,i+1}+t_{n,1}},$$

$P_{i,j}$ represents the probability of currently being located on the road between the *i*-th and *j*-th waypoints of the route. *n* represents the total number of waypoints, and $t_{i,j}$ is the time it takes to move between the *i*-th and *j*-th waypoints.

If the current location is determined using Eq. (8), the actual travel time from any point on the road to the test point is measured, and the same process is repeated for all test points.

In the first experiment, for all cases, we generated the routes using five coverage parameter values and compared them to each other. We check the first rank (optimal) parameter values having the smallest cumulative travel time and the second rank (second-best) parameter values (Table 3). In addition, the number of times each parameter value is selected as the first rank and the number of times each parameter value is selected as the second rank can be checked in (Table 4).

**Table 3:** First and second ranks of *coverage* parameter.

Section	Time Slot															
	Slot 1		Slot 2		Slot 3		Slot 4		Slot 5		Slot 6		Slot 7		Slot 8	
	Rank #1	Rank #2	Rank #1	Rank #2	Rank #1	Rank #2	Rank #1	Rank #2	Rank #1	Rank #2	Rank #1	Rank #2	Rank #1	Rank #2	Rank #1	Rank #2
1	0.5	0.7	0.5	0.0	1.0	0.7	0.0	0.3	1.0	0.7	0.5	0.0	0.7	0.5	0.0	1.0
2	0.3	1.0	0.7	0.3	0.7	0.3	0.7	0.0	0.7	1.0	1.0	0.0	0.5	0.3	0.0	0.7
3	0.5	0.3	1.0	0.7	1.0	0.3	0.3	0.5	0.0	0.3	1.0	0.7	0.0	1.0	0.5	0.3
4	0.3	0.7	1.0	0.0	0.5	0.0	0.0	0.7	0.5	0.0	0.3	0.5	0.5	0.7	0.7	0.3
5	0.5	0.3	0.5	0.7	0.7	1.0	0.5	0.7	0.3	0.0	0.7	0.3	0.5	1.0	1.0	0.7
6	0.5	0.7	1.0	0.0	0.0	0.7	0.3	0.0	1.0	0.3	1.0	0.0	1.0	0.3	0.3	0.5
7	1.0	0.7	0.5	1.0	1.0	0.7	1.0	0.5	1.0	0.0	1.0	0.7	0.0	0.7	1.0	0.3
8	1.0	0.3	1.0	0.0	0.5	0.7	1.0	0.7	0.5	0.3	1.0	0.3	0.5	0.3	0.3	0.7
9	1.0	0.7	0.7	0.0.	0.5	0.7	0.5	0.5	0.5	0.7	0.5	1.0	1.0	0.0	1.0	0.7

**Table 4:** Number of times *coverage* parameter is selected for the first and second ranks.

Optimal Parameters	coverage parameter value										Total Counts
	0.0		0.3		0.5		0.7		1.0		
	Count	Ratio	Count	Ratio	Count	Ratio	Count	Ratio	Count	Ratio	
The First Rank	8	0.11	8	0.11	21	0.29	10	0.14	**25**	**0.35**	72
The Second Rank	15	0.21	19	0.26	5	0.07	**25**	**0.35**	8	0.11	72

The parameter value most frequently selected as the first rank is 1.0 which accounts for 35% of all cases. Also, the second most selected parameter value was 0.5, accounting for 29% of all cases. The number of cases with an optimal coverage parameter value of 0.5 or higher is 78% of all cases. On the other hand, the number of cases with an optimal coverage value of 0.0 is selected the least as 11% of all cases. Also, the coverage parameter value most frequently selected as the second rank is 0.7 which accounts for 35% of all cases.

Based on the results, we have seen that considering hotspots, which we define as crime risk areas, in the generation of patrol routes is effective at preventing sporadic criminal incidents. As the coverage value increases, the probability of being close to a sporadic crime area increases because the route is optimized to pass through as many hotspots as possible. Therefore, we observed a reduction in the cumulative initial response time compared to routes with lower hotspot coverage.

In the second experiment, a comparative analysis was performed to evaluate the efficiency of our proposed method compared to the conventional patrol method. Therefore, we compared the performance of our proposed routes generated with optimized parameters to the performance of a random route that moves between randomly selected points in the shortest time as a control. To compare the cumulative initial response time for each route, response time is measured in the same way as in the coverage parameter optimization step. Five random routes for performance comparison were created for each case, and the travel time to 1000 test points was measured.

**Figure 13:**
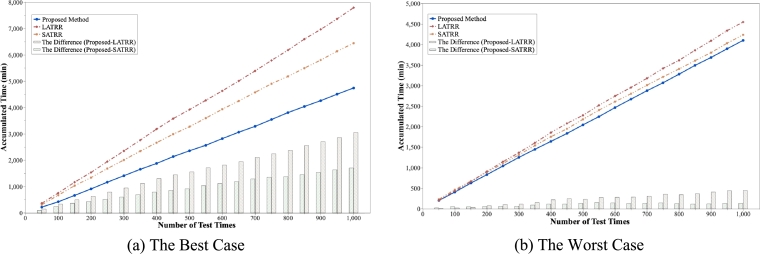
Experimental results. (a) shows the best case among all cases, and (b) shows the result of the worst case. The line graph shows the accumulated travel time of the routes as the number of test points increases. Each graph corresponds with the route of the proposed method, random route of the largest accumulated time (LATRR), and random route of the shortest accumulated time (SATRR), respectively. In addition, the bar graph shows the difference in the cumulative initial response time between the proposed method and each random route.

According to the experimental results, we find the case (time slot 2, section 7) that saved the most time on average among all 72 cases when compared with five random routes for the same case. We also find the worst case (time slot 1, section 2) that, on average, saved the least amount of time. The proposed algorithm can save a maximum of approximately 3050 minutes and a minimum of approximately 1701 minutes of travel time in the best case when 1000 crime cases occur (Fig. 13-a). In addition, in the worst case, it is possible to reduce the maximum travel time by approximately 445 minutes and to reduce the minimum by approximately 134 minutes (Fig. 13-b).

From these results, we proved that our proposed patrol routes can save anywhere from 134 minutes to 3050 minutes of time for 1000 randomly generated criminal incidents when using real-world navigation. When dealing with criminal cases, a slow response time can be fatal, so time savings corresponding to the experimental results can be very helpful in preventing actual criminal incidents. We also demonstrated that the proposed patrol route is effective in preventing and responding to potential crimes at multiple locations. We extracted hotspots by considering local properties and the correlation between crime and various community data. Additionally, we generated optimal routes to patrol as many crime-prone areas as possible. Therefore, if patrol personnel and patrol vehicles are deployed based on the proposed route, human and material resources can be saved. Additionally, since we proposed a generalized crime risk estimation and patrol route optimization algorithm, it is consistently applicable and scalable in regions with different data and geographical features.

## 5 Application

**Figure 14:**
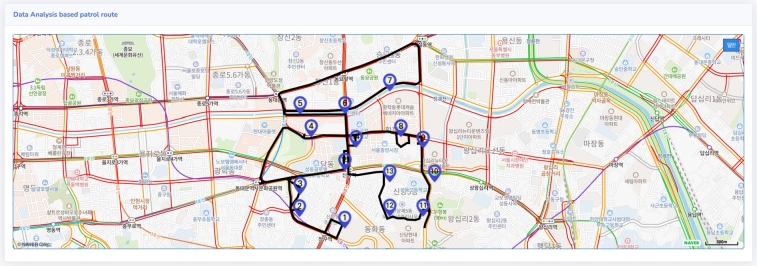
Patrol route recommendation application based on the computational optimization approach method. When the user selects the season, time, and section, the optimal patrol route reflecting real-time traffic information is provided.

In the model designed in this research, cases are determined by selecting the season, time, and section, and then the patrol route for the case is created. When the program is executed, a hotspot corresponding to the selected case is extracted, and based on this, the coordinates of the optimized route are transmitted to the Map API to create a patrol route that reflects real-time traffic information (Fig. 14).

## 6 Discussion

With the aim of designing a patrol system suited specifically to Korea, this study analyzed local community data and crime data to predict potential hotspots and suggested optimal patrol routes that could reduce the time needed to arrive at the site of an incident. Existing domestic patrol patterns predict hotspots based solely on confirmed criminal cases or define hotspots based on areas having the highest crime rates. In contrast, this study utilized data that are fundamentally different from emergency calls to develop an algorithm that could optimize police patrol routes. Most importantly, relying on unfiltered data from previous emergency calls to design patrol routes can raise issues related to data reliability, because atypical cases, such as repeated calls, duplicate incidents, and non-urgent civil complaints, are included. Herein lies the unique contribution of the analysis and model presented in this study.

Predictive police activities are a type of intervention that has received a lot of attention, and many countries have developed algorithms applying different statistical methodologies and deployed them in hotspot areas. Its use is now growing in other environments, especially in Western countries, such as COMPuter STATistics (COMPSTAT) in the New York City Police Department (NYPD) [45], PredPol in the Los Angeles Police Department (LAPD) [46], Domain Awareness System (DAS) in New York [47], Crime Prediction and Prevention by IBM Security Solutions [48], and the National Intelligence Model (NIM) in the United Kingdom [49]. Besides, many evaluation research results have reported the effectiveness of predictive policing in crime prevention. These predictive policing methods use algorithms that analyze numerous data, such as crime reports and arrest records, in order to predict hotspot areas by applying various statistical methods (e.g., hotspot mapping, time series analysis, correlation analysis, near-repeat model, etc.), but they have some usefulness and limitations.

This study is distinct from the geographic profiling analyses conducted by foreign police in the following ways. While the NYPD's COMPSTAT uses crime data to allocate personnel and establish policing strategies, this study is unique in that it integrates local statistics potentially relevant to crime, which allows for a correlation analysis to identify high-risk hotspots and a route optimization algorithm for effective police patrols. LAPD's predictive policing (PredPol) is also similar in that it predicts regional crime rates. However, PredPol's predictions of crime-prone areas are based on machine learning methods, and the learning cycle occurs every six months, hindering the immediate update of newer data [50]. In contrast, our method utilizes correlation analysis results between crime data and other relevant public data to predict crime hotspots, which allows for flexible additional analysis of newly collected or updated data. Moreover, the model incorporates real-time traffic information to generate up-to-date patrol routes.

Despite the meaningful contributions described above, this study faces the following limitations. In order to analyze the correlation between crime data and local community data, it is necessary to have accumulated data of actual crime. However, crime data is not available in South Korea for security purposes, and local community data that can be collected is limited. Therefore, if crime data and local community data are sufficiently provided through cooperation with local governments, more accurate crime risk evaluation will be possible through the method suggested in this study. Moreover, to analyze the performance of the proposed route optimization algorithm, information on patrol routes is needed for comparison. However, empirical research on algorithms that produce patrol routes based on crime data is lacking, and the actual patrol routes of law enforcement agencies are not open to public due to security reasons. As such, the performance was compared to routes that patrol locations extracted from crime data in random order during the verification stage. In the future, a database of real police patrol routes should be created, and by resolving these limitations, the effectiveness of this study can be proven in comparison to the actual routes implemented.

Nevertheless, this study has empirically validated the effectiveness of smart policing using hotspots and a route optimization algorithm for the first time in South Korea. Moreover, this study contributes to both the academic and public policy sectors by comparing existing domestic and foreign geographic profiling systems and proposing areas for improvement. Future benchmarking of this method, especially in countries where local crime data utilization is feasible, will be an encouraging step for international research on hotspots policing.

## CRediT authorship contribution statement

**Dongyeon Kim:** Software, Visualization, Writing – original draft, Writing – review & editing. **Yejin Kan:** Data curation, Investigation, Methodology. **YooJin Aum:** Data curation, Methodology. **Wanhee Lee:** Formal analysis, Methodology, Project administration, Resources. **Gangman Yi:** Conceptualization, Funding acquisition, Project administration, Supervision.

## Declaration of Competing Interest

The authors declare that they have no known competing financial interests or personal relationships that could have appeared to influence the work reported in this paper.

## Data Availability

Data associated with this study has been deposited at https://www.data.go.kr/index.do and https://data.seoul.go.kr.

## Footnotes

1 https://www.data.go.kr/index.do.

2 https://data.seoul.go.kr.

3 KOSIS; https://kosis.kr/index/index.do.

4 https://www.ngii.go.kr.
